# Characterization of *Kluyveromyces marxianus* Extracellular Vesicles as a New Potential Delivery System for Biologically Active Compounds

**DOI:** 10.3390/ijms27209259

**Published:** 2026-10-18

**Authors:** Marta Rogalska, Katarzyna Kmita, Anna Katarzyna Wrońska, Aleksander Gryciuk, Ewa Sitkiewicz, Magdalena Bakun, Magdalena Świadek, Małgorzata Milner-Krawczyk, Jolanta Mierzejewska

**Affiliations:** 1 Chair of Drug and Cosmetics Biotechnology, Faculty of Chemistry, Warsaw University of Technology, 00-664 Warsaw, Poland; marta.rogalska2.dokt@pw.edu.pl (M.R.); katarzyna.kmita.dokt@pw.edu.pl (K.K.); aleksander.gryciuk.dokt@pw.edu.pl (A.G.); malgorzata.krawczyk@pw.edu.pl (M.M.-K.); 2 Museum and Institute of Zoology, Polish Academy of Sciences, ul. Twarda 51/55, 00-818 Warsaw, Poland; awronska@miiz.waw.pl; 3 Mass Spectrometry Laboratory, Institute of Biochemistry and Biophysics, Polish Academy of Sciences, 02-106 Warsaw, Poland; ewa@ibb.waw.pl (E.S.); bakunm@ibb.waw.pl (M.B.); 4 Laboratory of Electron Microscopy, Nencki Institute of Experimental Biology, Polish Academy of Sciences, 02-093 Warsaw, Poland; m.swiadek@nencki.edu.pl

**Keywords:** probiotic-derived EVs, yeast, *Galleria mellonella*, biocompatibility, immunomodulation, doxorubicin loading

## Abstract

Extracellular vesicles (EVs) released by probiotic microorganisms may be involved in intercellular communication via transferring their cargo to other cells. Hence, there is growing interest in their application as a drug delivery system. This article presents studies on EVs secreted by three strains of *Kluyveromyces marxianus* (B0399, WUT240, and WUT216) under standard growth conditions and in fluids simulating a gastrointestinal environment. Nanoparticle tracking analysis and transmission electron microscopy revealed spherical EVs with modal diameters ranging from 79.8 to 148.2 nm, depending on the growth conditions. Research conducted on human intestinal cell lines did not reveal cytotoxic effects of EVs. Moreover, EVs exhibited mild antioxidant properties in normal cells. *K. marxianus* EVs did not stimulate the release of the pro-inflammatory cytokine IL-8 in the HT-29 model but induced a tailored immunomodulatory response in *Galleria mellonella* hemocytes, characterized by elevated levels of IL-8-like proteins in the absence of a TNF-α-like response. Proteomic analysis identified 161 core proteins common to all strains, yielding 33 potential biomarkers lacking significant human homology, which would be useful for tracking yeast EV carriers in subsequent studies. In addition, yeast EVs were found to have a high affinity for human cells, transporting biologically active molecules (doxorubicin) and the Nile Red dye into recipient cells. Altogether, this study provides clear evidence that the EVs of *K. marxianus* could be considered a drug delivery system.

## 1. Introduction

The concept of probiotics has evolved beyond the World Health Organization definition and now encompasses their potential use as carriers of biologically active compounds [1,2,3]. A significant line of research focuses on the transport of nanoparticles, proteins, and antigens by probiotic bacteria and yeasts. This live drug delivery system offers several advantages, such as biocompatibility, low toxicity, and the ability to interact with host tissues. However, there are some limitations, such as susceptibility to antibacterial or antifungal drugs, and some concerns such as the potential to cause bloodstream infections among patients with weakened immune systems [3,4,5].

Research into extracellular vesicles (EVs) may challenge these problems as an innovative non-live drug delivery system. Previous studies have shown that EVs released by probiotic bacteria and yeasts can interact with other cells and positively influence immune modulation, metabolic disorders, gut health, and microbial and viral infection in human cell line models in vitro and in animal models in vivo [6,7,8,9]. Moreover, there is growing evidence that EVs of non-pathogenic yeast are not toxic to normal human intestinal cell lines in vitro and exhibit systemic biocompatibility in vivo in wax moths, *Galleria mellonella*, which are a well-established invertebrate model for the preliminary assessment of whole-organism toxicity and the innate immune system [7,8,9,10]. However, despite increasing interest in the biotechnological potential of EVs, there are still technical challenges that limit their use in clinical practice, such as variations in their composition and yield associated with different isolation methods, the presence of factors and contaminants in the preparation, and difficulties in finding suitable preclinical models for research [6]. Therefore, a detailed characterization of EVs is necessary before exploring their potential applications.

Recent reports indicate that some *Kluyveromyces marxianus* strains exhibit probiotic properties, including strong antioxidant activity and the ability to inhibit the growth of *Candida albicans*, as well as molds belonging to the *Aspergillus* and *Fusarium* genera [11,12]. They can also modulate the host’s immune response [12]. In addition, for patients with irritable bowel syndrome, these yeasts, in combination with *Bifidobacterium animalis subsp. lactis*, alleviated symptoms without causing any significant changes in the human intestinal microbiota [1,12]. Since live *K. marxianus* is frequently consumed by humans, for example, in kefir, it is likely that after ingestion, yeast can interact with intestinal cells via its EVs. However, there is still a lack of evidence to support this hypothesis.

The aim of this study was to investigate whether *K. marxianus*-derived EVs transport biologically active molecules into human intestinal cells in vitro. Three well-known strains—B0399 (a commercial probiotic), WUT240, and WUT216 (isolated from fermented mare’s and cow’s milk)—were selected for this purpose [11]. Initially, the research focused on the comprehensive characterization of EVs produced by *K. marxianus* strains cultured in both standard medium and fluids simulating the human gastrointestinal environment (stomach, small intestine, and colon). This was achieved using nanoparticle tracking analysis (NTA), transmission electron microscopy (TEM), and proteomic profiling to identify specific EV biomarkers. Furthermore, the safety profile of *K. marxianus* EVs was evaluated in detail, with a particular focus on their impact on metabolic activity and reactive oxygen species (ROS) levels in human intestinal cell lines. To this end, a panel of five cell lines was used, comprising both normal intestinal epithelial cells and their tumor counterparts. This approach enabled the simultaneous assessment of the safety and biocompatibility of EVs with regard to healthy cells, as well as determination of whether they exhibit selective activity against cancer cells. Analysis of metabolic activity and ROS levels also allowed the verification of whether any anti-tumor activity might be associated with the induction of oxidative stress in cancer cells. Additionally, immunogenicity was assessed by monitoring the production of pro-inflammatory proteins in the HT-29 in vitro model and in the hemocytes of *G. mellonella* larvae. Finally, to confirm the hypothesis that yeast EVs can serve as a delivery system, a two-step validation process was conducted. This comprised the cellular uptake of Nile Red-labeled EVs and the targeted delivery of doxorubicin into human cells.

## 2. Results

### 2.1. EV Characterization

**Figure 1:**
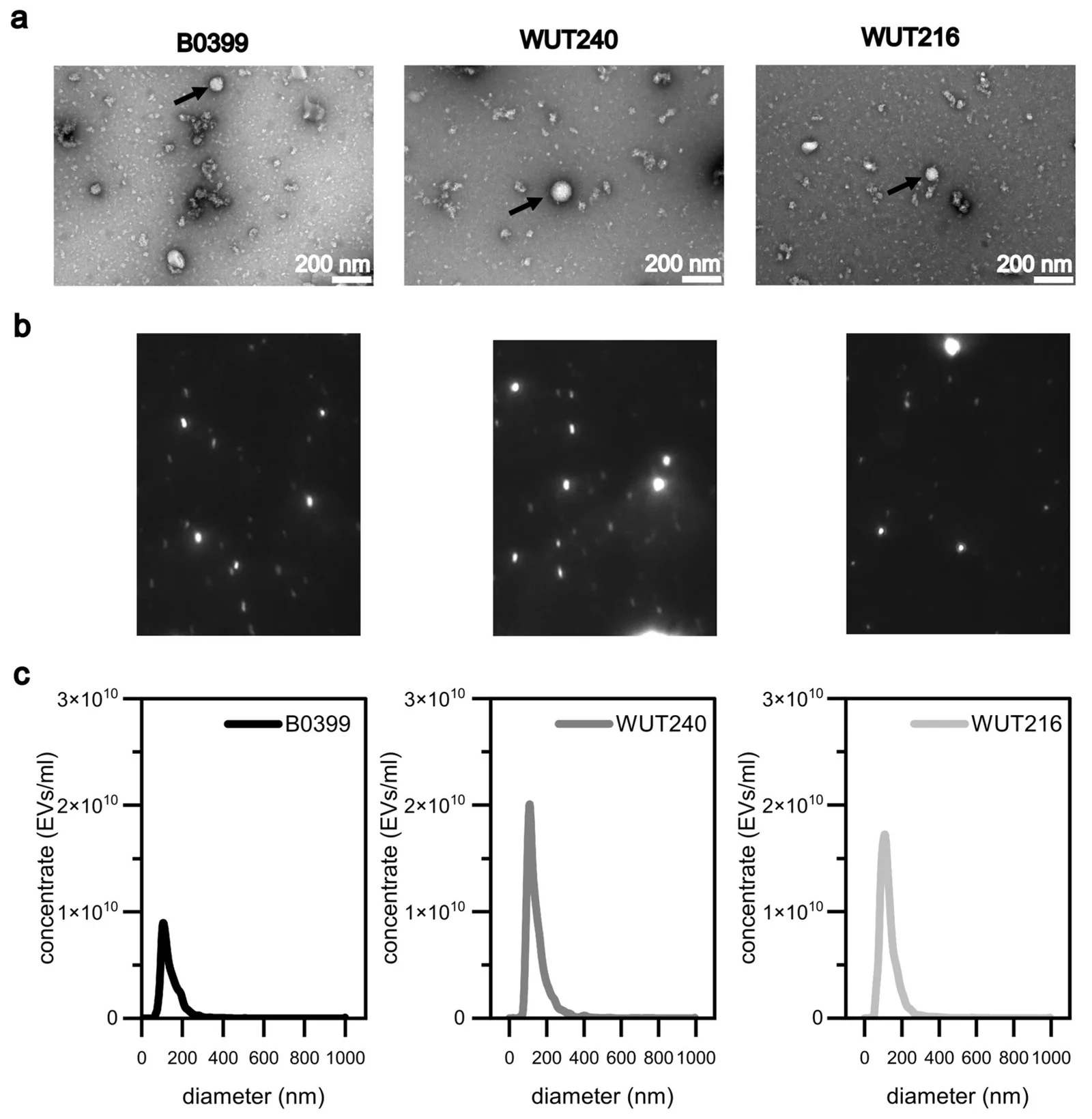
Physical characteristics of EVs produced by *K. marxianus* B0399, WUT240, and WUT216 strains. (**a**) TEM images (black arrows indicate representative vesicles). NTA (**b**) images in fluorescence mode and (**c**) size distribution (*n* = 3).

The yeast cultures were grown in SAB liquid medium, which is routinely used for cultivation of microscopic fungi. Concentrated fractions containing EVs were then obtained from the culture supernatant. The morphology of EVs was subsequently imaged using TEM, which revealed spherical structures with a distinct dark staining pattern, indirectly indicating the presence of a lipid bilayer (Figure 1a). Furthermore, staining of yeast-derived EVs with the DiO dye, followed by NTA in fluorescence mode, confirmed the presence of membrane structures in the samples (Figure 1b).

Size distribution and concentration of the isolated EVs were determined using NTA in bright-field mode (Figure 1c). The modal diameters of EVs released by *K. marxianus* B0399, WUT240 and WUT216 were 107.5 ± 2.39 nm, 115.2 ± 5.98 nm and 97.3 ± 2.49 nm, respectively. The mean diameters of EVs from B0399, WUT240 and WUT216 strains were 144.3 ± 2.55 nm, 148.2 ± 2.76 nm and 136.0 ± 3.61 nm, respectively. Thus, the WUT240 strain produced the largest particles under the tested conditions. The concentration of EVs in 1 mL of yeast cultures in SAB medium was as follows: 1.08 × 10^10^ ± 1.85 × 10^9^ EVs for B0399, 2.01 × 10^10^ ± 7.11 × 10^9^ EVs for WUT240, and 5.01 × 10^10^ ± 2.07 × 10^10^ EVs for WUT216.

**Figure 2:**
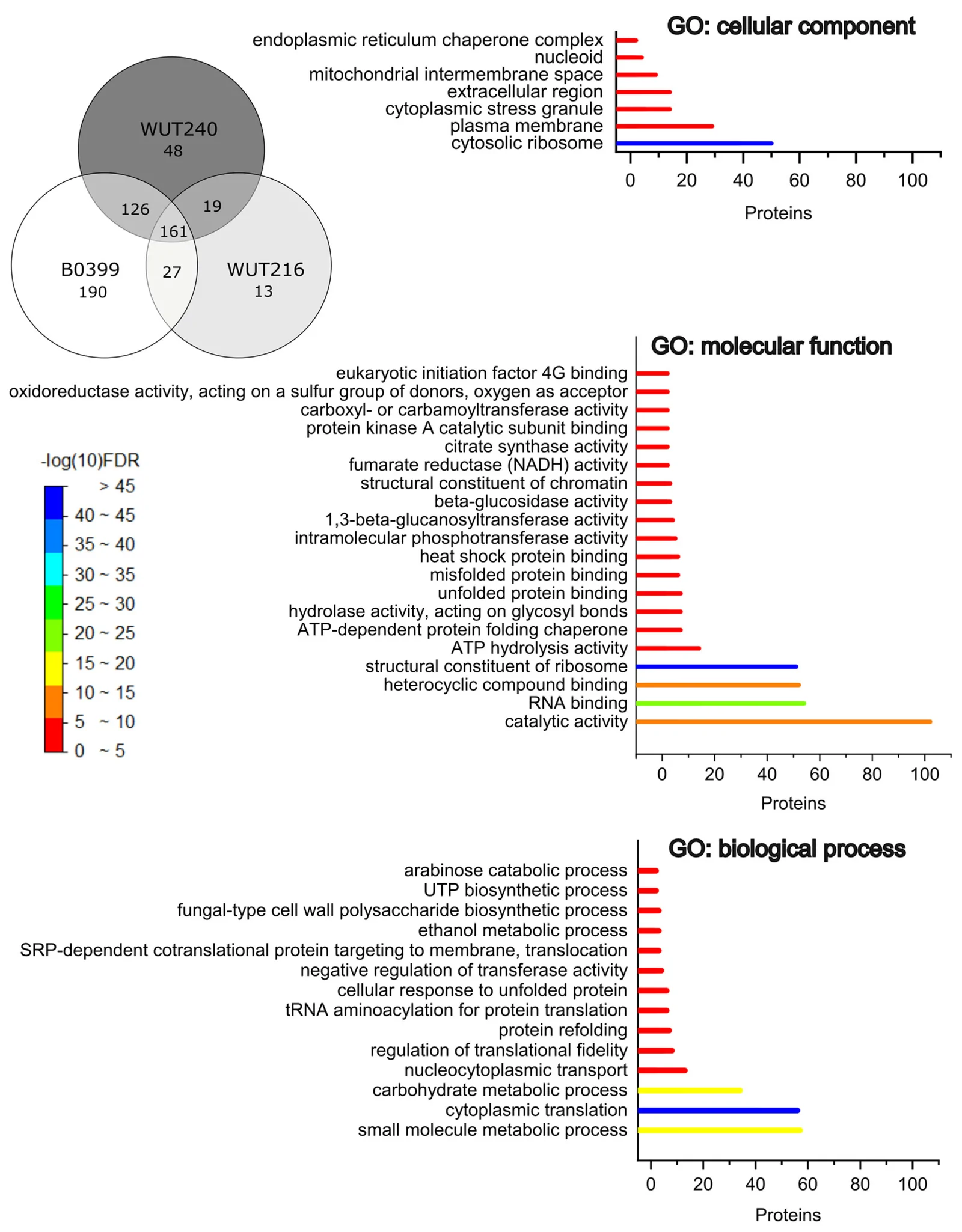
Proteomic analysis of *K. marxianus* EVs. Proteins shared by three strains (B0399, WUT240 and WUT216)—Venn diagram and GO enrichment of common proteins analysis of cellular components, molecular functions, and biological processes (*n* = 3).

It is well known that cells secrete protein-rich EVs into their environment, which may potentially influence the activity of other cells upon contact. Therefore, in the next step of this study, a proteomic analysis was carried out to examine the proteins present in the EVs of three strains of *K. marxianus* in detail. The protein composition of yeast EVs was analyzed using mass spectrometry, and the list of identified proteins is available in the Supplementary Materials (Tables S1–S3). According to the NCBI database for *K. marxianus*, 504 proteins were identified in B0399’s EVs, 354 in WUT240’s EVs, and 220 in WUT216’s EVs; each was represented by at least three peptides across three biological replicates. Of these, 190 were unique to B0399’s EVs, 48 to WUT240, and 13 to WUT216 (Figure 2 and Table S4). The pool of proteins common to the three strains totaled 161, which was subjected to further proteomic analysis to determine the proteins that were most frequently present in *K. marxianus* EVs. Enrichment of the cellular component gene ontology was performed (Figure 2). Among these, as many as 50 proteins (~31%) belonged to the cytoplasmic ribosome (FDR < 10^−40^). Another large group, accounting for around 18% of the total, consisted of cell membrane proteins (*N* = 29, FDR < 10^−4^). Furthermore, 14 proteins belonging to the extracellular region (~9%, FDR < 10^−6^) and cytoplasmic stress granules (FDR < 10^−7^) were identified. GO functional enrichment analysis revealed that approximately 35% of the proteins were associated with cytoplasmic translation (*N* = 56, FDR < 10^−43^) and the small-molecule metabolic process (*N* = 57, FDR < 10^−13^). A significant number of the proteins were also involved in carbohydrate metabolism (*N* = 34, FDR < 10^−13^). The GO molecular function analysis assigned approximately 63% of the proteins to catalytic activity (*N* = 102, FDR < 10^−7^) (Figure 2). The following functions could be distinguished: RNA binding (*N* = 54, FDR < 10^−15^), heterocyclic compound binding (*N* = 52, FDR < 10^−7^), and structural constituents of ribosomes (*N* = 51, FDR < 10^−34^).

In addition, attempts were made to identify protein biomarkers that not only occur in all EV populations derived from the studied *K. marxianus* strains but also do not show significant homology with human proteins. This list may be valuable in future studies aimed at monitoring the localization of *K. marxianus* proteins during direct contact between yeast EVs and human cells using immunological techniques. Therefore, a key selection criterion was to prevent potential cross-reactivity and ensure the specific detection and localization of proteins contained in yeast EVs within human cells.

A total of 33 potential biomarkers were selected from the population of common proteins based on the calculated range (Table S5). Several of these are involved in carbohydrate metabolism (Hxk1, Lac4, Inu1, Icl1, Mls1, Pck1, Xyl1-2 and Aaap1), cell wall remodeling (Exg1, Gas1, Gas3, SCW4, SUN4, SIM1 and Crh2) and the formation of eisosomes (Pil1 and Lsp1). It is worth noting that the selected biomarkers also include proteins whose function is either unknown or based solely on hypotheses.

### 2.2. Evaluation of EV Production in Media Simulating Human Gastrointestinal Fluids

Previous studies have shown that strains WUT216 and WUT240, like B0399, exhibit significant survival in environments mimicking gastrointestinal fluid in vitro [11]. The objective was therefore to examine the efficiency of EV secretion by *K. marxianus* under these harsh conditions.

The B0399, WUT240, and WUT216 strains were incubated in SGF, SIF, and SCF for 1, 2, and 24 h, respectively, to simulate the conditions they encounter when passing through the human digestive tract. Then, EVs were harvested from the cultures, and their size distribution and concentration were determined using NTA (Table 1). This analysis revealed no significant differences in EV size distribution between samples incubated in SGF, SIF, and SCF and their respective controls (yeast cells suspended in SAB-based liquid). However, in most cases, the EVs collected from gut fluids were slightly larger (mean diameter).

**Figure 3:**
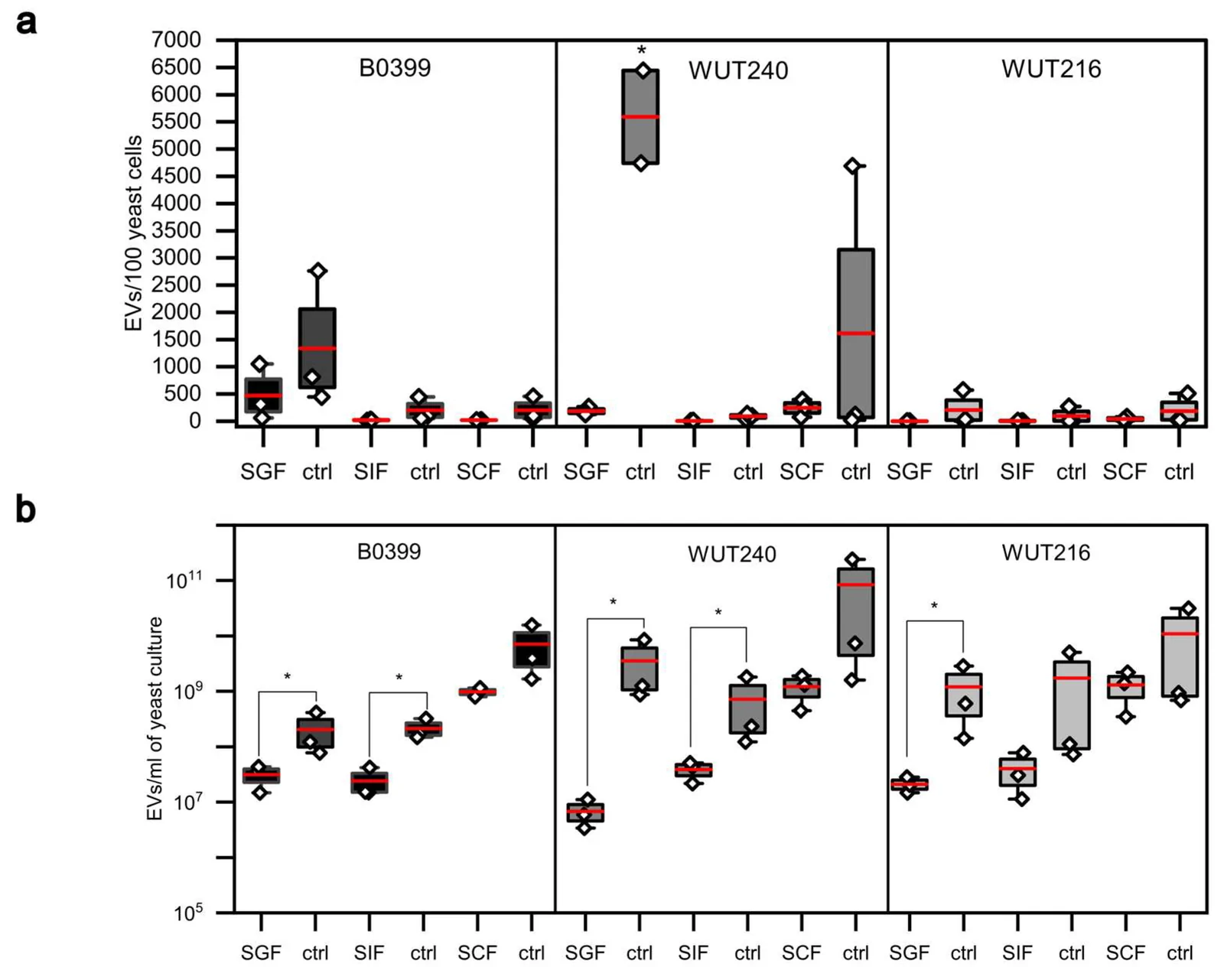
Extracellular vesicle secretion by three strains of *K. marxianus* (B0399, WUT240 and WUT216) in fluids mimicking the gastrointestinal tract environment (SGF, SIF, and SCF). For each culture variant, a corresponding control in SAB-based medium was used (ctrl). (**a**) Specific EV productivity distribution (EVs/100 yeast cells); only the control in SGF in the set of WUT240 cultures differs significantly from others (mean ± SE, *n* = 3, one-way ANOVA with a Tukey HSD post hoc test, * *p* < 0.05). (**b**) Total EV output (EVs/mL of yeast culture); data transformed to log10 prior to statistical analysis due to a large variance and differences in magnitude (mean ± SE, *n* = 3, one-way ANOVA with Tukey’s HSD post hoc test, * *p* < 0.05). The red line indicates the mean values.

To evaluate the efficiency of vesicle release under different conditions, specific EV productivity was calculated and expressed as EVs per 100 yeast cells (Figure 3a). The overall concentration of vesicles obtained from the culture broth was also determined, and this was expressed as volumetric EV yield (EVs/mL of culture) (Figure 3b). With one exception, exposure of *K. marxianus* strains to artificial digestive fluids (SGF, SIF, and SCF) did not result in any statistically significant changes in EV-specific productivity compared to the corresponding controls. Nevertheless, a trend emerges whereby environments mimicking gastrointestinal conditions slightly reduce EV productivity. In nearly every case, the mean values calculated for gut fluids are lower than those of their respective control cultures. Broad comparisons across strains revealed that the secretory profiles of B0399, WUT240 and WUT216 are consistent with one another and do not differ significantly within the same media and incubation times. The only deviation from this pattern was observed in the control of the WUT240 culture in SGF, where a sudden, statistically significant release of extracellular vesicles was observed. Importantly, this effect disappears completely in the presence of gastric juice (SGF).

In contrast to specific cellular EV productivity, analysis of the total yield of vesicles per 1 mL of the initial culture revealed clearly visible differences between environments mimicking the gastrointestinal tract and control conditions. Exposure to gastric fluids (SGF, 1 h) and intestinal fluids (SIF, 2 h) resulted in a significant decrease in the total number of released EVs for strains B0399 and WUT240 compared to their control counterparts (*p* < 0.05). A similar trend of inhibition of total secretion under the influence of gastric juice was also observed for strain WUT216. Noteworthy, after 24 h of incubation in colonic fluid (SCF), the total yield of EVs approached control levels for all three strains.

### 2.3. The Functional Effects of EVs on Normal and Cancerous Human Cell Lines

Another aspect of the study involved investigating whether *K. marxianus* EVs can affect both cancer and normal human intestinal epithelial cells in vitro. Cell–EV interactions were evaluated using MTT and ROS assays to assess metabolic activity (MA) and measure oxidative stress. EVs collected from yeast cultures in SAB and simulated colonic fluid (SCF) were selected for those tests due to their high recovery yield.

**Figure 4:**
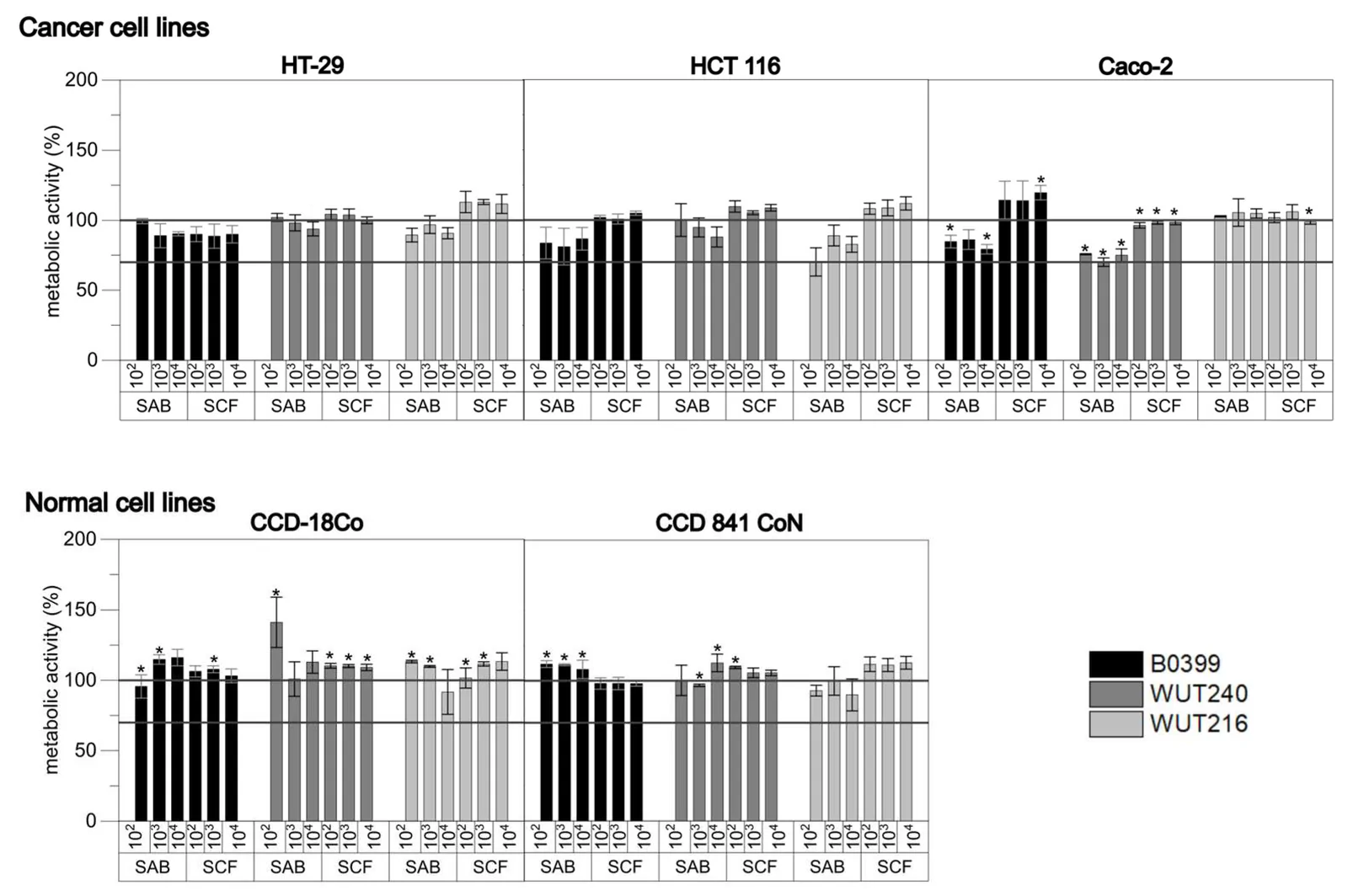
Metabolic activity (MA) of human intestinal cell lines (HT-29, HCT 116, Caco-2, CCD-18Co and CCD 841 CoN) after treatment with *K. marxianus*’ EVs at conc. of 10^2^–10^4^ EVs/cell. EVs originated from the cultures of B0399, WUT240, and WUT216 strains in SAB and SCF media. MA values of the positive control (5% DMSO) were as follows: 27.0 ± 1.34%, 9.8 ± 1.65%, 5.9 ± 2.69%, 19.1 ± 2.78%, and 41.7 ± 2.05% for the HT-29, HCT 116, Caco-2, CCD-18Co, and CCD 841 CoN cell lines, respectively. Each tested variant was compared with the nontreated control cells (100%) and presented as a percent of the control activity level. Mean values ± SE (*n* = 3); * indicates statistical significance in comparison with the nontreated controls, measured by one-way ANOVA with Tukey’s HSD post hoc test, *p*-value < 0.05. The top line indicates the level of nontreated controls (100%), and the bottom line indicates the level of cytotoxicity (70%) in accordance with ISO standard [13].

When evaluating the metabolic activity (MTT assay; Figure 4) of intestinal cell lines after 24 h treatment with yeast EVs, no clear dose-dependent relationship was observed within the tested concentration range of 10^2^–10^4^ EVs/cell. Negative effects of these particles were observed across all tested cell lines and EV production environments. However, a significant negative effect on MA was primarily observed in cancer cell lines, notably Caco-2 and HCT 116, exposed to EVs isolated from yeast cultures in SAB medium. However, this reduction stayed above the usual 70% viability threshold that is generally accepted as the standard for determining whether something is cytotoxic [13]. In contrast, EVs derived from yeast cultures in SCF medium generally maintained or slightly enhanced MA in cancer cell lines. A statistically significant stimulatory effect on MA was observed in several treatments in normal cell lines (CCD-18Co and CCD 841 CoN), particularly for EVs produced by the B0399 strain in SAB medium and WUT240 and WUT216 in SCF medium. Nevertheless, the magnitude of these increases was modest (~10–25%), suggesting limited biological impact.

**Figure 5:**
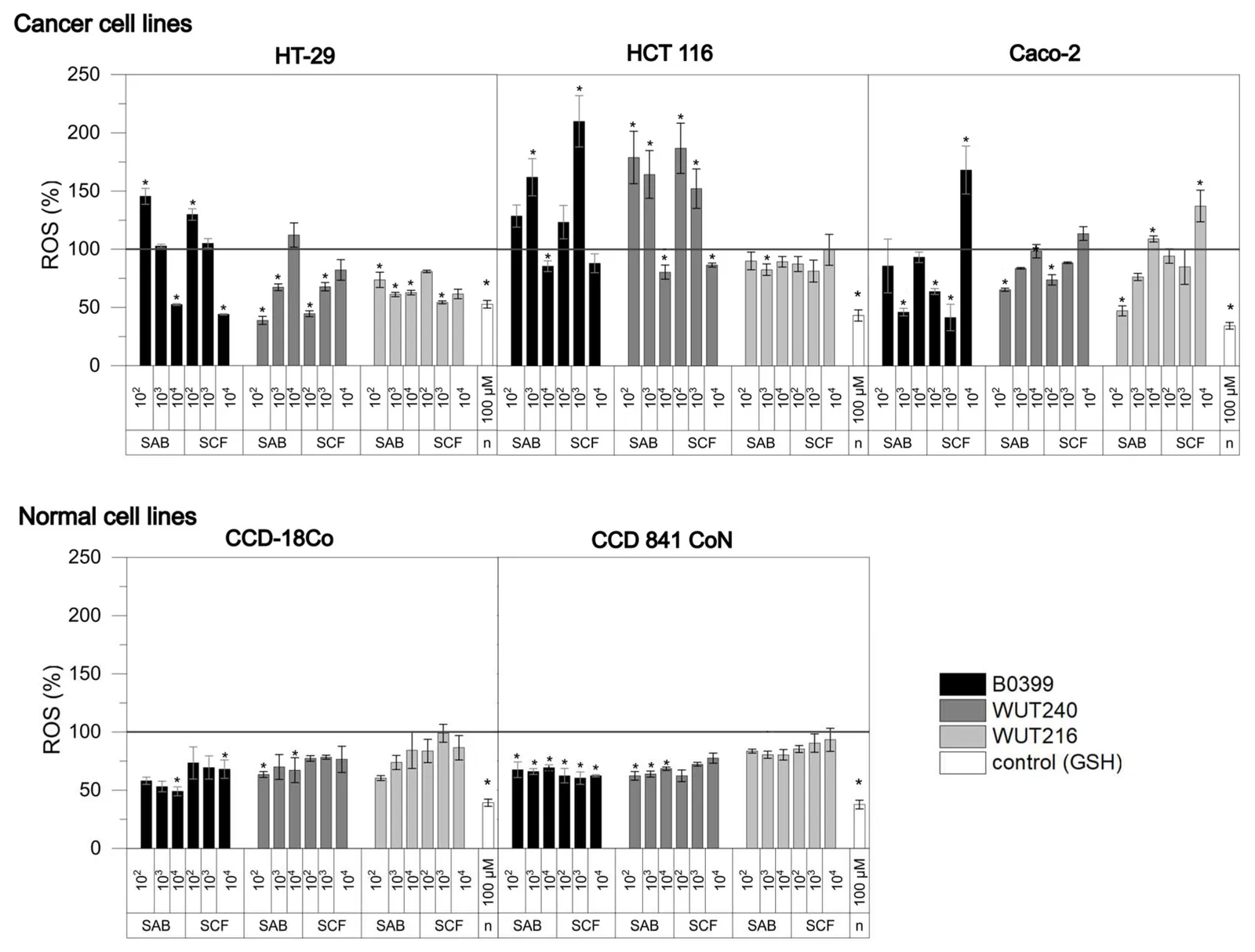
ROS levels in human cancer (HT-29, HCT 116 and Caco-2) and normal (CCD-18Co and CCD 841 CoN) intestinal cell lines after treatment with *K. marxianus* EVs at conc. of 10^2^–10^4^ EVs/cell. EVs originated from cultures of B0399, WUT240, and WUT216 strains in SAB and SCF media. “*n*” means negative control (GSH). t-BHP was used as a positive control and increased ROS levels to 2001.2 ± 123.67%, 1296.1 ± 268.72%, 836.93 ± 193.70%, 1880.7 ± 164.87%, 1587.1 ± 267.54% in cells HT-29, HCT 116, Caco-2, CCD-18Co and CCD 841 CoN, respectively. Each tested variant was compared with the untreated control cells (100% indicated by the line) and presented as a percent of the control activity level. Mean values ± SE (*n* = 3); * statistical significance in comparison with the untreated controls measured by one-way ANOVA with Tukey’s HSD post hoc test, *p*-value < 0.05.

The release of ROS induced by yeast vesicles was largely independent of the production environment (SAB or SCF medium). The effect of EVs on healthy cells was clearly distinct from that observed in cancer cells. The presence of EVs had no adverse or pro-oxidative effect on normal human cells. In fact, they consistently exhibited an antioxidant effect, reducing ROS levels below the control threshold in a manner similar to GSH (see Figure 5). However, no clear dose-dependent relationship was observed in this case. The effect of yeast EVs on cancer cells was much less consistent and varied depending on the cell line and the source of the vesicles. Specifically, in the HCT 116 cell line, EVs from strains B0399 and WUT240 exhibited marked pro-oxidant activity at lower concentrations (10^2^–10^3^), elevating ROS levels by up to ~130–210%, whereas WUT216 EVs exerted a slight antioxidant effect. In the HT-29 and Caco-2 cell lines, EVs from strain B0399 generally exhibited an antioxidant trend at most concentrations; however, this shifted towards pro-oxidant action at the lowest dose in HT-29 cells and the highest dose in SCF-grown Caco-2 cells. A marked antioxidant effect of EVs from both WUT strains was also observed in HT-29 cells. However, in the case of WUT216 EVs, no clear dose-dependent trend was evident. These results suggest that, while *K. marxianus* EVs act as safe antioxidants in normal intestinal cells, they have a highly strain- and cell-line-dependent impact on cancer cells, occasionally inducing strong pro-oxidant responses.

The HT-29 cell line is often used in studies investigating the immune response to postbiotics and probiotics because it can secrete cytokines in response to inflammatory stimuli [14,15,16]. The effect of yeast EVs on the production of the pro-inflammatory cytokine IL-8 was therefore assessed using this cell line. The release of IL-8 was quantified in cell culture supernatants after a 24 h exposure to yeast EVs. IL-8 levels were as follows (mean values ± SE; *n* = 3): 170.83 ± 3.82 pg/mL, 171.51 ± 3.50 pg/mL and 173.19 ± 3.25 pg/mL in the cultures treated with B0399, WUT240, and WUT216 EVs, respectively. A similar IL-8 concentration (174.19 ± 2.42 pg/mL) was observed in the negative control (untreated with EVs). Meanwhile, IL-8 secretion was significantly enhanced (222.63 ± 2.70 pg/mL) in the positive control incubated with LPS (*p* < 0.05). Thus, treating human HT-29 cells with yeast EVs did not increase IL-8 release.

### 2.4. Immunolocalization of IL-8-like and TNF-α-like Proteins in G. mellonella Hemocytes Exposed to K. marxianus EVs

**Figure 6:**
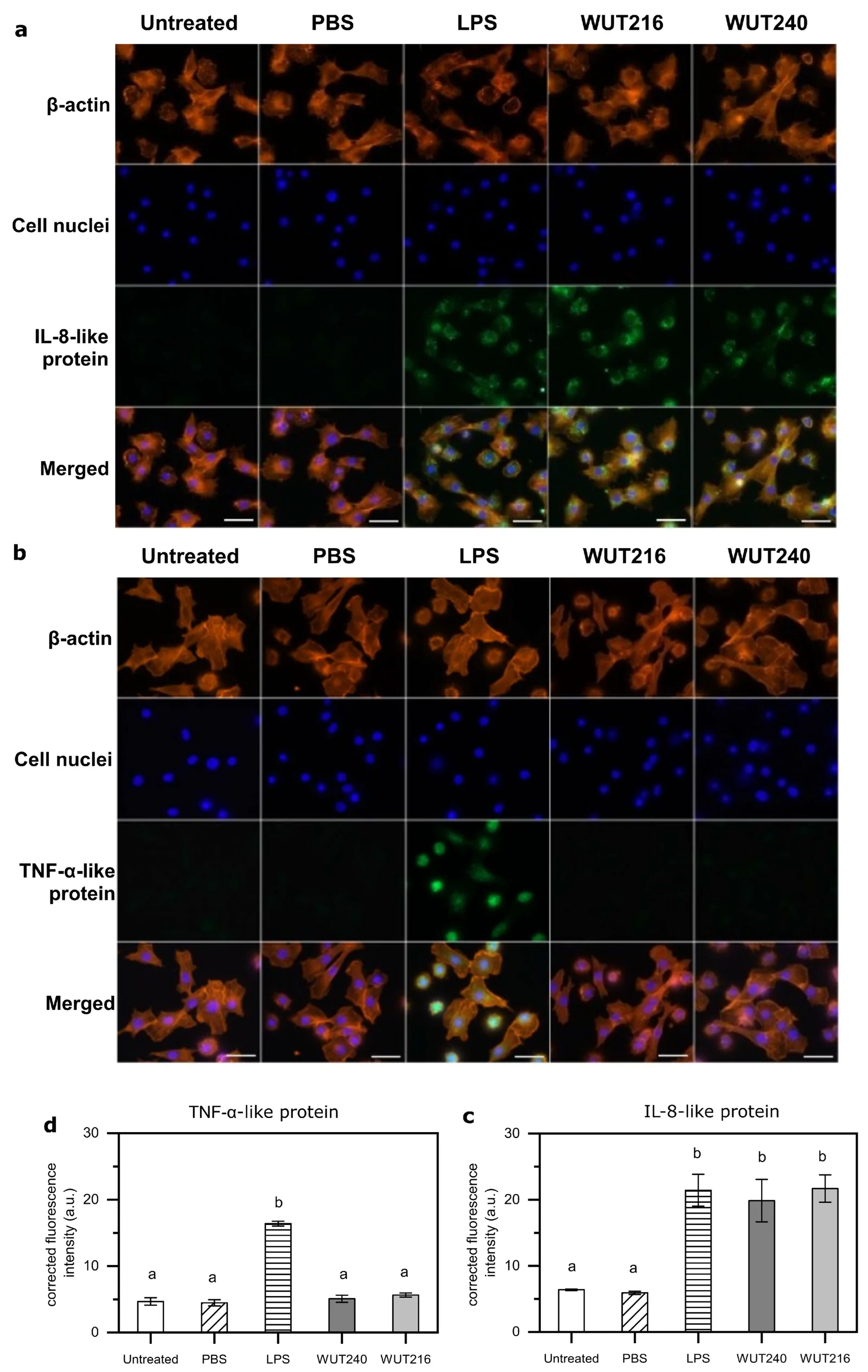
Immunofluorescence staining and quantification of IL-8-like (**a**,**c**) and TNF-α-like (**b**,**d**) protein levels in *G. mellonella* hemocytes following treatment with *K. marxianus*-derived EVs. (**a**,**b**) β-actin (orange) was stained with ActinRed 555 ReadyProbes Reagent. Cell nuclei (blue) were stained with NucBlue™ Fixed Cell ReadyProbes™ Reagent. IL-8-like and TNF-α-like proteins (green) were detected using rabbit polyclonal primary antibodies and a goat anti-rabbit IgG (H + L) DyLight 488 secondary antibody. The images demonstrate untreated cells, hemocytes treated with PBS, hemocytes stimulated with LPS (100 ng/mL), and hemocytes treated with EVs from the *K. marxianus* WUT216 and WUT240 strains, respectively; the scale bar is 25 μm. (**c**,**d**) For each image, the mean fluorescence intensity of the green channel was measured and corrected by subtracting the mean background intensity measured in a cell-free region of the same image. Corrected values were expressed in arbitrary units (a.u.). Groups labeled with different letters differed significantly from one another; groups sharing the same letter did not differ significantly (one-way ANOVA with Tukey’s HSD post hoc test, *p* < 0.05, mean values ± SD, *n* = 3).

To assess whether *K. marxianus*-derived EVs affect the immune response of *G. mellonella* hemocytes, the presence of IL-8-like and TNF-α-like proteins was examined by immunofluorescence staining and quantified by measuring corrected fluorescence intensity in the green channel (Figure 6). Raw fluorescence intensity measurements for all images and replicates are provided in the Supplementary Materials (Table S6). Across all experimental groups, β-actin and cell-nuclei staining confirmed the presence of intact, adherent hemocytes, indicating that neither LPS nor EV treatment affected overall cell morphology or attachment under the tested conditions (Figure 6a,b).

For the IL-8-like protein (Figure 6a,c), little to no green fluorescence signal was observed in untreated or PBS-treated hemocytes, where staining was limited to a faint, diffuse background. A distinct, cytoplasmic, green signal was clearly visible in hemocytes stimulated with LPS, as well as in hemocytes treated with EVs derived from either *K. marxianus* strain WUT216 or WUT240; in both EV-treated groups, the signal appeared visually comparable in distribution and intensity to that observed in the LPS-stimulated positive control. This impression was confirmed by quantitative image analysis: corrected fluorescence intensity was low in untreated (6.4 ± 0.1 a.u.) and PBS-treated (5.9 ± 0.3 a.u.) groups and increased markedly (*p* < 0.0001) in LPS (21.4 ± 2.4 a.u.), WUT216 (21.7 ± 2.1 a.u.), and WUT240 (19.9 ± 3.2 a.u.) probes. Tukey’s HSD post hoc test confirmed that LPS, WUT216, and WUT240 were each significantly higher than untreated and PBS (*p* < 0.001 for all six comparisons), whereas no significant difference was found among LPS, WUT216, and WUT240 themselves (*p* > 0.05 for all pairwise comparisons). Together, the microscopic and quantitative data indicate that EVs from both *K. marxianus* strains increased the detection level of the IL-8-like protein in *G. mellonella* hemocytes to an extent indistinguishable from that elicited by LPS.

In contrast, for the TNF-α-like protein (Figure 6b,d), a distinct green signal, concentrated mainly in the perinuclear region of the cells, was observed almost exclusively in the LPS-stimulated group; hemocytes from the untreated, PBS-treated, and EV-treated (WUT216 and WUT240) groups showed only faint, background-level staining that was visually indistinguishable from the negative controls. Quantitative image analysis supported this observation: corrected fluorescence intensity remained low and statistically comparable among untreated (4.7 ± 0.6 a.u.), PBS-treated (4.5 ± 0.5 a.u.), WUT216 (5.7 ± 0.3 a.u.), and WUT240 (5.1 ± 0.5 a.u.) (*p* > 0.05 for all pairwise comparisons among these four groups) samples, whereas the LPS-treated (16.4 ± 0.4 a.u.) group was significantly higher than all other groups (*p* < 0.0001 vs. each of the remaining four groups). Together, these observations indicate that, unlike LPS, *K. marxianus*-derived EVs did not visibly or statistically increase the detection level of the TNF-α-like protein in *G. mellonella* hemocytes under the tested conditions.

Taken together, both the qualitative microscopic observations and the quantitative fluorescence intensity analysis indicate that *K. marxianus* EVs selectively increase the detection level of the IL-8-like but not the TNF-α-like protein in *G. mellonella* hemocytes, in contrast to LPS, which increased the detection level of both cytokine-like proteins. Full ANOVA and Tukey’s HSD post hoc statistics for both cytokines are reported in Supplementary Materials (Table S6).

### 2.5. Fluorescence Imaging of the Interaction Between Yeast EVs and Human Intestinal Cell Lines

**Figure 7:**
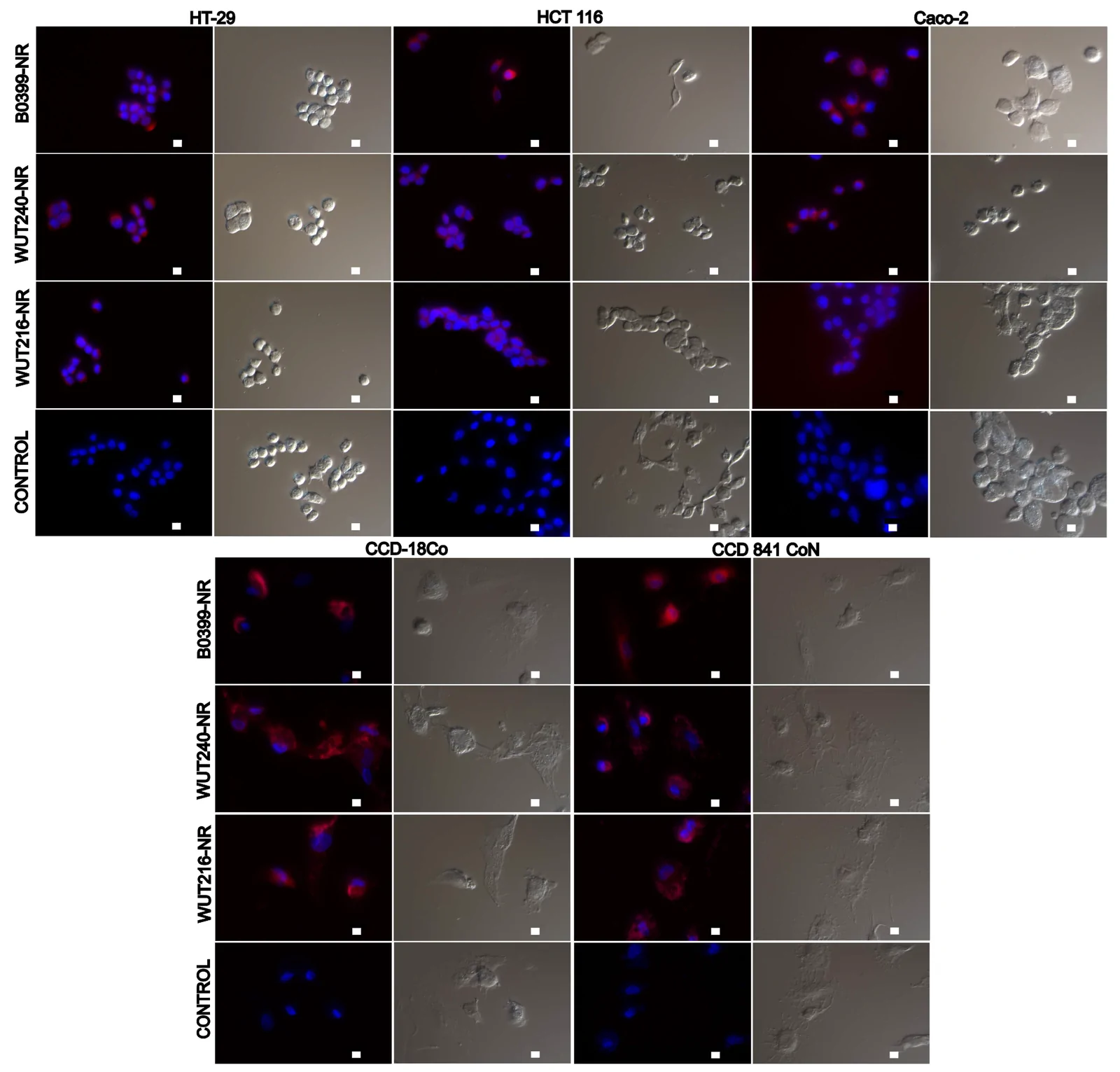
Representative fluorescence microscopy images illustrating interaction of *K. marxianus* EVs with human cancer (HT-29, HCT 116, and Caco-2) and normal (CCD-18Co and CCD 841 CoN) cell lines. B0399-NR, WUT240-NR, and WUT216-NR—yeast EVs collected from cultures of the B0399, WUT240, and WUT216 strains in SAB and stained with Nile Red dye (red fluorescence) prior to being added to cells. Control—PBS sample lacking EVs subjected to identical staining and washing steps. Cellular nuclei were stained with DAPI (blue fluorescence). The scale bar is 10 µm.

The next step of the study involved evaluating the direct interaction between the yeast EVs and human cells in vitro using fluorescence microscopy. The EVs, which originated from the yeast cultures in SAB and SCF, were stained with the lipophilic dye Nile Red (NR) prior to being added to normal and cancerous colon epithelial cells for fifteen minutes. Qualitative microscopic observations revealed a clearly visible NR fluorescence signal associated with the cells (see Figure 7 and Figure S1; red fluorescence). This suggests that the labeled EVs interact strongly with, or are internalized by, the human cell lines. Importantly, the control showed no red fluorescence, confirming that the free dye had been completely removed from the EVs.

### 2.6. Fluorescence Microscopy Evidence of Doxorubicin Delivery to Human Cells via Yeast EVs

**Figure 8:**
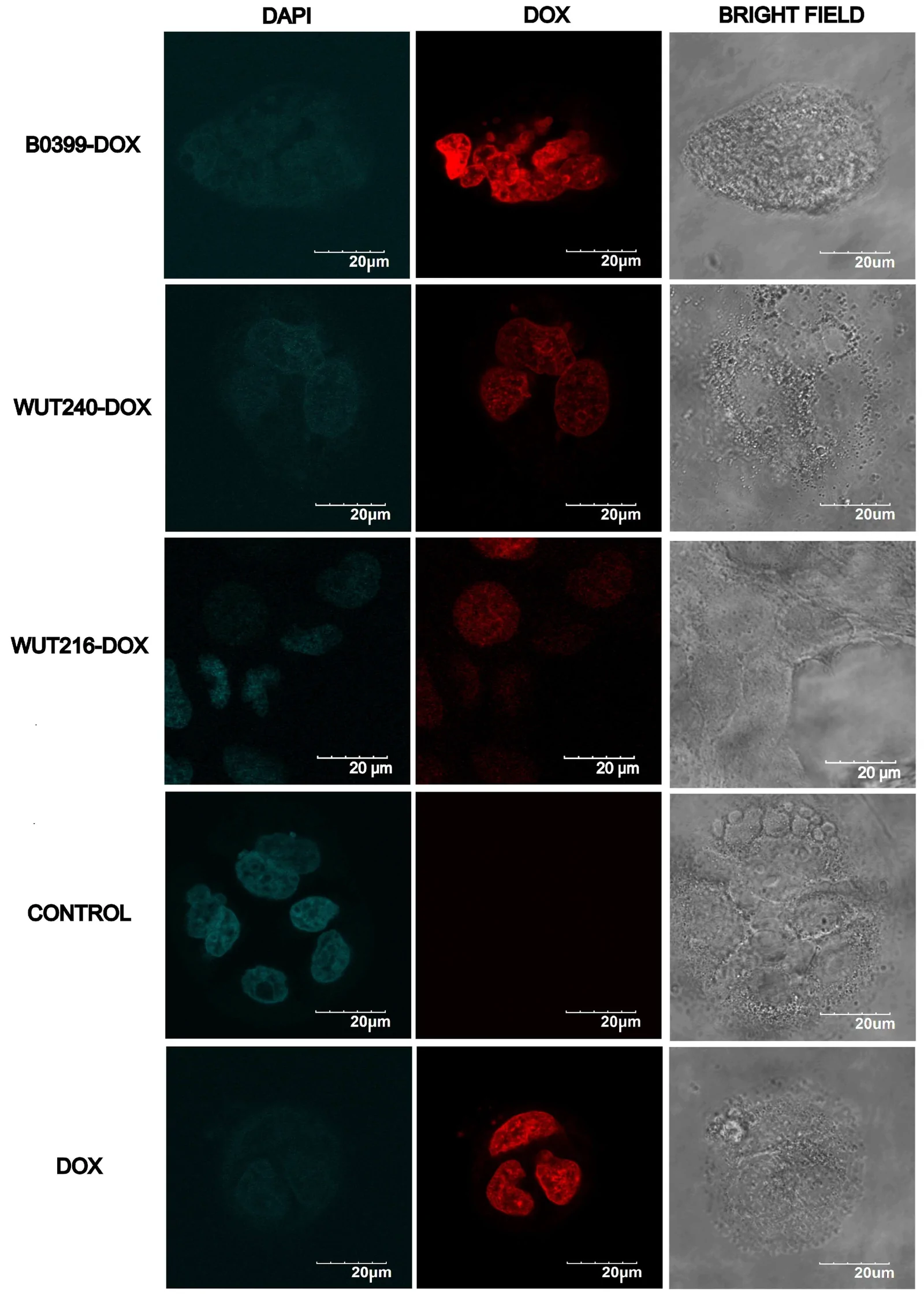
Representative fluorescence microscopy images demonstrating the transfer of doxorubicin (DOX; red fluorescence) to human Caco-2 cells via yeast EVs. B0399-DOX, WUT240-DOX and WUT216-DOX—the EVs of B0399, WUT240 and WUT216 strains, respectively, loaded with doxorubicin. Control—PBS sample. DOX—“free” doxorubicin in PBS solution. Caco-2 nuclei were stained with DAPI.

Knowing that *K. marxianus* EVs can interact directly with human cells, the ability of these vesicles to deliver the hydrophilic anticancer agent doxorubicin to Caco-2 cells was then investigated. Following one hour of incubation with either free doxorubicin (DOX) or doxorubicin encapsulated in EVs (B3099-DOX, WUT240-DOX, and WUT216-DOX), distinct accumulation of the drug within the cell nuclei was observed (see Figure 8 for red fluorescence). Noticeably, the presence of doxorubicin in the nuclei reduced the efficiency of DAPI intercalation into DNA. This was evident from the weaker blue fluorescence intensity observed in cells treated with doxorubicin, both “free” and loaded in EVs, compared to the control (Table S7).

## 3. Discussion

Extracellular vesicles have been recognized as mediators of intercellular communication, and consequently, there is a growing interest in their potential use as carriers of biologically active substances. While the most advanced research focuses on EVs derived from human cells, scientists have also recently turned their attention to probiotic microorganisms, particularly lactic acid bacteria [17,18]. Nevertheless, several recent reports have been published on EVs derived from the well-established probiotic *S. cerevisiae var. boulardii* strain and from potentially probiotic *S. cerevisiae* strains [8,9,10,14,15,16,19], as well as from *Pichia fermentans* [19], and *Metschnikowia pulcherrima* [20]. Among yeast species considered as probiotic candidates is *K. marxianus*, one of the most extensively studied in this context, being the B0399 strain [12]. Additionally, this yeast species exhibits many valuable biotechnological traits, such as rapid growth on a wide range of carbon sources, making its secretory activity and EVs worthy of closer examination. The only previous publication on *K. marxianus* EVs showed that, based on the in vitro studies, these vesicles could be considered postbiotics for preventing *Candida albicans* vaginal infections [7].

The present study focused on a different aspect: the potential of *K. marxianus* EVs to act as a delivery system into human cells, a finding that had not been reported previously. To this end, three strains—B0399, WUT240 and WUT216—were used. Firstly, a detailed characterization of EVs derived from yeast cultures in SAB medium was conducted. TEM imaging of EV samples revealed structures similar to those described in previous studies on other yeast species [9,10,19].

When considering the use of probiotic yeast and its EVs as carriers of biologically active substances, it is important to examine these particles in detail and evaluate the efficiency of their secretion. The size, quantity, and composition of EVs are currently known to be determined not only by species and culture conditions but also by strain [21]. The results presented herein also revealed some differences in EV size within *K. marxianus* species. The WUT240 strain produced the largest particles, while the WUT216 strain produced the smallest vesicles when growing in the SAB medium. The mean diameter of the EVs obtained in this study (approx. 136.8–148.2 nm) is slightly lower than the 157.7 nm recently reported for *K. marxianus* grown in YPD medium [7]. According to data from the literature on other yeast species, the average diameter of EVs is most commonly in the range of 85–212 nm, e.g., [8,9,19,20,22]. Therefore, *K. marxianus* vesicles are moderate in size.

The present findings also revealed variations in EV concentrations among three *K. marxianus* strains cultured in SAB medium. Specifically, the WUT240 and WUT216 strains produced a higher quantity of EVs (2.01 × 10^10^ ± 7.11 × 10^9^ and 5.01 × 10^10^ ± 2.07 × 10^10^ EVs/mL, respectively) than B0399 (1.08 × 10^10^ ± 1.85 × 10^9^ EVs/mL). Interestingly, *K. marxianus* appears to produce a higher volumetric concentration of EVs compared to previously reported titers for *Saccharomyces* strains, such as *S. cerevisiae var. boulardii* CNCM I-745 (3.47 ± 0.55 × 10^9^ EVs/mL) and *S. cerevisiae* WUT3 and WUT151 (4.35 ± 2.51 × 10^9^ and 3.72 ± 0.98 × 10^9^ EVs/mL), which were grown in SAB medium [9]. This is probably due to the faster growth rates of *K. marxianus* compared to *S. cerevisiae*. However, without cell density data from the earlier study, it is currently impossible to distinguish the effect of biomass from that of the actual EVs/cell ratio without conducting further comprehensive, parallel trials for all strains.

It is also important to look closely at the proteins that yeast send out in EVs when thinking about these vesicles as ways to carry biological substances. This approach can expand our knowledge of yeast secretory activity and identify marker proteins, which could facilitate the planning of genetic modifications to strains to secrete desired heterologous proteins with therapeutic effects on human cells via EVs. A comparative proteomic analysis was therefore conducted in the present study. This revealed conserved functional motifs and significant strain-dependent diversity within *K. marxianus* EV proteins. While our analysis identified a core set of 161 proteins common to all three strains, only 36 of these were found in the EV proteome recently described by Imparato et al. [7]. This limited overlap in the proteomes highlights the high degree of heterogeneity in EV cargo composition among strains, which may result from genetic variations, specific culture conditions, or the methodology of proteomic analysis. It should be noted here that the *K. marxianus* proteome has not yet been well studied, which limits the identification of proteins in this yeast’s EVs. Nevertheless, at the functional level, our results are similar to the observations of Imparato et al. [7]. Notably, both studies indicate that EVs from *K. marxianus* carry a protein cargo that is targeted at key metabolic processes, including carbohydrate metabolism. Another feature that links the two proteomes is the presence of enzymes involved in cell wall remodeling and degradation. This is reflected in the molecular function profile (e.g., the activity of beta-glucosidases and 1,3-beta-glucan transferases). Glucan 1,3-beta-glucosidase (Exg1), for example, was identified in both the vesicles we studied and the *K. marxianus* EV proteome described by Imparato et al. [7], confirming its conserved nature in the vesicles of this species. This enzyme is also present in EVs from other yeasts, including *S. cerevisiae* var. *boulardii* [10]. Furthermore, in both studies, the profile of cellular components indicates a significant contribution from proteins located in surface structures (membrane and cell wall) and in the extracellular space. However, it is worth noting that the EV cargo of the *K. marxianus* strains that we analyzed has a very high representation of ribosomal proteins (making up ~31% of the total proteome) and translational processes. The presence of such a large number of translational apparatus components, including RNA-binding proteins, cytosolic translation factors, and ribosomal structural components, suggests that these vesicles may modulate gene expression or protein synthesis in target cells.

Most importantly, this study expands upon the work of Imparato et al. [7] by presenting a meticulously selected set of 33 potential EV biomarkers. These biomarkers were chosen due to their lack of homology with human proteins. Seven of these were also present in the EVs described by Imparato et al. (Met6, Hxk1, Inu1, Scw4, Gas1, Gas4, and Sim1). These proteins are primarily involved in cell wall organization and remodeling, carbohydrate metabolism, methionine biosynthesis, and protein folding. This panel addresses a key methodological challenge in this field, offering a reliable tool for tracking the internalization of yeast EVs and the release of their cargo in human cell models, free from the risk of cross-reactivity.

This study, for the first time, also examined whether *K. marxianus* strains can secrete EVs under adverse conditions simulating human digestive fluids (SGF, SIF, and SCF) and how such conditions affect the size of the vesicles. The culture conditions accounted for appropriate ion and enzyme concentrations, pH, and incubation time to replicate physiological conditions as closely as possible. The survival of the analyzed strains has currently been demonstrated in these fluids [11]. The present report indicates that exposing yeast to simulated digestive conditions generally did not significantly influence the size profiles of the EVs. Furthermore, at the single-cell level, exposure to artificial digestive fluids (SGF, SIF and SCF) did not significantly affect the EV-specific productivity (EVs/cell). However, the slight overall downward trend in mean productivity in an environment simulating gastrointestinal conditions suggests that the cellular secretory apparatus remains functional in the presence of external stressors. Furthermore, the homogeneity of the productivity profiles of strains B0399, WUT240 and WUT216 indicates that this stable baseline release of vesicles is a constant, species-specific trait of *K. marxianus*, independent of the isolate’s environmental origin. However, when converting from cellular productivity to total EV yield per milliliter of culture, the differences between the digestive fluids and the control conditions became more pronounced and were generally statistically significant. For strains B0399 and WUT240, fluids that simulated gastric and intestinal conditions (SGF and SIF) caused a significant decrease in the total number of EVs recovered. This discrepancy with the cellular productivity data suggests that the reduction in the total pool of EVs is not due to impaired secretion mechanisms but rather to reduced yeast survival and lower biomass accumulation in the gastrointestinal environment. Interestingly, after a 24 h incubation in simulated colonic fluid (SCF), the total EV yield approached control levels for all evaluated strains. This may suggest that biomass is restored over a sufficiently long incubation period. It is worth noting that evaluating fully independent biological replicates inherently involves greater variability between batches. This explains why subtle changes in EV-specific productivity were not statistically significant. Nevertheless, the consistent decrease in total EV yield per milliliter of culture under SGF/SIF conditions, followed by recovery under SCF conditions, confirms that these trends reflect a genuine physiological phenomenon in all three strains.

Further steps were taken to investigate the effect of *K. marxianus* EVs on the metabolic activity and redox balance of both cancer and normal human colonic epithelial cells in vitro. An evaluation of the activity of *K. marxianus* EVs on various intestinal cell-line models demonstrated a favorable safety profile for the vesicles, with no apparent dose-dependent toxicity. Although EVs derived from yeast cultures in SAB overall had a slight inhibitory effect on the metabolic activity of colorectal cancer cell lines (Caco-2 and HCT 116), cell viability remained consistently above the 70% threshold required to rule out cytotoxic effects (EN ISO 10993-5:2009). Interestingly, cancer cells responded more mildly to EVs produced under simulated colon conditions (SCF)—the cells fully maintained or slightly enhanced their metabolism. In the case of non-cancerous intestinal cells (CCD-18Co and CCD 841 CoN), EVs—in particular, B0399 grown in SAB and WUT strains grown under SCF conditions—induced statistically significant metabolic stimulation. However, given that this increase ranged only from 10% to 25%, its broader physiological impact is likely to be minimal. Similar results were reported by Imparato et al. [7], who demonstrated that extracellular vesicles isolated from a *K. marxianus* strain were completely safe in vivo in a *G. mellonella* larval model and did not exert a cytotoxic effect on human vaginal epithelial cells (A-431 line). The consistent confirmation of the absence of toxicity across such diverse systems—ranging from epithelial models derived from various tissues to the invertebrate tissue model *G. mellonella*—validates the biological safety of *K. marxianus* vesicles.

It is well known that a critical level of reactive oxygen species (ROS) can damage host cells, proteins, lipids, and DNA. Therefore, it is essential to assess the effect of the test factor on the redox balance [23]. EVs from *K. marxianus* exhibited a significant functional difference when cultured with normal and cancerous intestinal cell lines. In healthy human cells, EVs consistently exerted a protective antioxidant effect, reducing ROS levels to a degree comparable to that of GSH, with no signs of pro-oxidative cellular toxicity. In contrast, their effects on colorectal cancer models were highly variable and depended strongly on the yeast strain and specific recipient cell line. Notably, in HCT 116 cells, EVs derived from strains B0399 and WUT240 induced a significant, temporary pro-oxidative response at lower doses (10^2^–10^3^). In contrast, in HT-29 and Caco-2 models, antioxidant effects predominated, and ROS induction occurred less frequently, only under specific experimental conditions. However, it should be noted that the maximum ROS values obtained were significantly lower than those induced by t-BHP. The ability to enhance oxidative stress in specific cancer states is intriguing but requires further research into the underlying mechanisms. More significant, however, is the fact that EVs from *K. marxianus* may act as antioxidants in the intact intestinal epithelium. This confirms their high safety profile and justifies further investigation into their role in protecting the gut against oxidative stress, which numerous studies have identified as a key factor in the pathogenesis of the most common gastrointestinal diseases [24]. Direct assessment of ROS modulation by EVs from probiotic yeasts is still rare in the literature. However, our observations are consistent with recent reports on EVs from *Pichia kudriavzevii*, which have also been shown to alleviate oxidative stress in intestinal epithelial models [25].

In order to develop a delivery system for active biomolecules to human cells using yeast EVs, it is necessary to consider the immunogenicity of these particles. The pro-inflammatory chemokine IL-8 recruits immune cells to sites of bacterial and fungal infection, and it is a key marker of the immune response in intestinal models, including the HT-29 cell line [26]. The present study of *K. marxianus* EVs revealed no induction of IL-8 secretion in HT-29 cells, with IL-8 levels remaining significantly lower than those observed following pro-inflammatory agent LPS stimulation. This suggests that the vesicles themselves do not induce an undesirable pro-inflammatory response in intestinal epithelial cells in vitro. Notably, Imparato et al. [7] recently demonstrated that *K. marxianus* EVs can inhibit IL-8 release induced by *C. albicans* in A-431 vaginal epithelial cells. However, they did not confirm whether *K. marxianus* EVs alone can influence IL-8 production by the A-431 cells. Thus, further research is needed to verify these findings in epithelial cells. This is particularly important given that studies on *Saccharomyces*-derived EVs have revealed different immunological responses in various in vitro models. For example, THP-1, human monocyte cells, increased their secretion of IL-8 and IL-1β following 24 h treatment with *S. cerevisiae* var. *boulardii* CNCM I-745 yeast EVs [10]. It has also been demonstrated that *S. cerevisiae* EVs stimulate the production of TNF-α and IL-6 in mouse macrophages (RAW264.7) and dendritic cells (DC2.4), respectively [14]. Another report showed that EVs derived from yeast isolated from fermented beverages can modulate immune system function by interacting with dendritic cells, thereby alleviating excessive inflammation [19]. To address these inconsistencies, the current study examined the impact of *K. marxianus* EVs on the immune response using hemocytes from *G. mellonella* larvae, another model for investigating interactions between microorganisms and host [27]. The presence of yeast vesicles was found to stimulate the production of IL-8-like protein, while TNF-α-like protein levels remained unchanged. Interestingly, following exposure of the hemocytes to LPS, levels of both pro-inflammatory proteins increased in these cells. This suggests that *K. marxianus* EVs have a distinct and selective immunomodulatory effect rather than triggering the generalized pro-inflammatory cascade typically elicited by potent bacterial endotoxins, such as LPS. Nevertheless, further in-depth research in this field is required, including the use of well-defined cell lines in in vitro studies and in vivo models.

Finally, regarding the potential use of probiotic yeast EVs as a drug delivery system, the interaction of *K. marxianus* EVs with human colon cells in vitro was investigated. The results presented here revealed that Nile Red-labeled *K. marxianus* EVs interact strongly with, or are internalized by, human cell lines within fifteen minutes of exposure. This highlights the high affinity of EVs for human epithelial cells. These rapid interaction kinetics are consistent with recent reports on *Saccharomyces*-derived EVs [9], suggesting that efficient contact with recipient cell membranes may be a common feature of yeast-derived EVs. Although membrane dye accumulation alone does not allow for an unambiguous distinction between surface binding and complete internalization, the successful delivery of doxorubicin (DOX) to the cell nucleus within one hour provides functional evidence of efficient endocytosis and intracellular cargo release. In addition, cells exposed to free or EV-encapsulated DOX exhibited an apparent reduction in DAPI fluorescence intensity compared to untreated controls. This is consistent with previously reported interactions between nuclear anthracyclines and DNA-binding fluorophores [28]. Similar observations of DOX delivery to human cells in vitro have also been reported for *S. cerevisiae* var. *boulardii* CNCM I-745 [8].

Taken together, these findings provide evidence that *K. marxianus* EVs could be a promising natural delivery system for therapeutic applications. However, further in-depth research is required, particularly with regard to their potential use in the oral delivery of biologically active substances.

## 4. Materials and Methods

### 4.1. Yeast Strains

*K. marxianus* WUT216 and WUT240 strains were isolated from dairy products as reported previously [29], and strain B0399 originates from the commercial Italian probiotic supplement DiarYeast (LongLife, Milano, Italy). The WUT yeasts are part of the Warsaw University of Technology Yeast Collection (https://www.wutyeastcollection.pw.edu.pl/ (accessed on 20 August 2026)) and stored as glycerol stock at −80 °C. Yeasts were revived by seeding onto SAB agar (Sabouraud dextrose broth with 2% agar, Biomaxima, Lublin, Poland) via spreading a sample taken directly from a frozen glycerol stock (strains WUT216 and WUT240) or a DiarYeast sample (B0399). The plates were then incubated at 37 °C for 24 h. Single colonies were subsequently used to inoculate the appropriate liquid media.

### 4.2. Isolation of EVs from Liquid Yeast Cultures

EVs were isolated from yeast cultures in SAB according to the previously described protocol [8]. First, 7 mL of SAB (Sabouraud dextrose broth, Biomaxima, Lublin, Poland) was inoculated with a single colony of yeast and incubated overnight at 37 °C with shaking at 240 rpm (Benchtop shaker SI-600R LabCompanion, Billerica, MA, USA). Then, 2.5 mL of the resulting culture was added to 200 mL of fresh SAB and incubated under the same conditions. After incubation, the cells were harvested by centrifugation (6000× *g*, 10 min, RT), and the supernatant was filtered using a 0.2 µm sterile PES vacuum filter (VWR, Radnor, PA, USA). The permeate fractions were concentrated in 15 mL portions using the 100 kDa regenerated cellulose centrifugal filters (Merck, Darmstadt, Germany) and spinning at 3200× *g* for 8 min at 4 °C. The concentrated EVs were collected in a sterile centrifugal flask each time. After that, the EVs were washed with 15 mL of phosphate-buffered saline (PBS) without calcium or magnesium (VWR International, Leuven, Belgium) and filtered using the same membrane. EVs were stored at −80 °C.

In order to isolate EVs secreted by yeasts during incubation in fluids mimicking the gastrointestinal tract environment, 200 mL cultures were first prepared in SAB as described above, subsequently centrifuged at 2900× *g* for 10 min at room temperature (RT), and the cell pellets were resuspended in 200 mL of sterile water. The 75 mL portions of the suspension were mixed with 75 mL of the simulated gastrointestinal fluids or SAB (served as a control) and incubated as detailed below [11]. EVs were then isolated from the fluids as described above.

Simulated Gastric Fluid (SGF): First, 0.5 mg/mL KCl (Chempur, Piekary Śląskie, Poland), 0.1 mg/mL KH_2_PO_4_ (Pol-Aura, Dywity, Poland), 2.1 mg/mL NaHCO_3_ (Chempur, Piekary Śląskie, Poland), 2.5 mg/mL NaCl (Chempur, Piekary Śląskie, Poland), 0.02 mg/mL MgCl_2_ (Chempur, Piekary Śląskie, Poland), 0.04 mg/mL (NH_4_)HCO_3_ (Merck, Darmstadt, Germany), 0.02 mg/mL CaCl_2_(H_2_O)_2_ (Chempur, Piekary Śląskie, Poland) and 4000 U/mL pepsin (Merck, Darmstadt, Germany) were dissolved in the sterile tap water. The mixture was adjusted to a pH of 3.0. The incubation parameters were as follows: 1 h of incubation at 37 °C with shaking at 100 rpm.

Simulated Small Intestinal Fluid (SIF): In this step, 0.5 mg/mL KCl, 0.1 mg/mL KH_2_PO_4_, 7.1 mg/mL NaHCO_3_, 2.2 mg/mL NaCl, 0.07 mg/mL MgCl_2_, 3.0 mg/mL CaCl_2_(H_2_O)_2_, 2.0 mg/mL pancreatin (Merck, Darmstadt, Germany), and 5 mg/mL of bile salts (Merck, Darmstadt, Germany) were dissolved in sterile tap water. The mixture was adjusted to a pH of 7.0. The incubation parameters were: 2 h of incubation at 37 °C with shaking at 100 rpm.

Simulated Colon Fluid (SCF): Next, 0.2 mg/mL KCl, 0.24 mg/mL KH_2_PO_4_, 1.44 mg/mL NaHCO_3_, 8.0 mg/mL NaCl, and 30.0 mg/mL SAB were dissolved in sterile tap water. The mixture was adjusted to pH 7.0. The incubation parameters were as follows: 24 h at 37 °C with shaking at 220 rpm.

Additionally, after incubation, 20 µL samples were collected from each culture condition (SAB, SGF, SIF, and SCF), diluted tenfold, and plated onto SAB agar plates. After 24 h of incubation at 37 °C, the number of visible colonies was counted to determine the number of colony-forming units (CFU/mL).

### 4.3. Size Distribution and Morphological Assessment of EVs

The size distribution and concentration of the EVs were analyzed using Nanoparticle Tracking Analysis (NTA; NanoSight Pro, Malvern Panalytical, Malvern, UK) with NS Explorer software version 1.1.0.6, with automatic camera and focus settings and a flow rate of 5 µL/min, capturing 10 videos of 750 frames each. Prior to measurement, samples were diluted in PBS at ratios of 1:200 or 1:1000 (*v*:*v*) for EVs collected from 1 h and 2 h cultures or from 24 h cultures, respectively. The samples were stained with 0.4 µg/mL of DiO (3,3′-dioctadecyloxacarbocyanine perchlorate, Merck, Darmstadt, Germany) and incubated in the dark for 30 min at RT.

The morphology of EVs was analyzed via transmission electron microscopy (TEM) at the Laboratory of Electron Microscopy at the Nencki Institute of Experimental Biology (PAS), which is part of the Polish Euro-BioImaging Node, employing the procedure documented previously in Gryciuk et al. [9].

### 4.4. Proteomic Profiling of EVs

Protein identification was performed at the Mass Spectrometry Laboratory at the Institute of Biochemistry and Biophysics PAS (Warsaw, Poland), as previously described [9]. Obtained peptide masses and fragmentation spectra were matched against the NCBI database restricted to *K. marxianus* (15,588 sequences). The proteomics data were deposited at https://repository.jpostdb.org/mypage (accessed on 26 August 2026) under project number JPST004863 [30] and are available via ProteomeXchange with dataset identifier PXD082951.

The list of identified proteins was exported to MS Excel and cleaned by removing hits that occurred in fewer than three biological replicates, as well as each query that had fewer than three peptides. Next, protein IDs were matched to the associated protein names or locus tags using the NCBI database. Only entries with assigned protein names were retained. Because the gene ontology (GO) tools require systematic gene names as input, the protein names were matched with the corresponding *S. cerevisiae* S288C systematic names using the UniProt Swiss-Prot database. Then, GO enrichment (cellular component, molecular function, and biological process) and KEGG pathway analysis were performed using g:Profiler, with *S. cerevisiae* S288C as the reference species. The data were analyzed using the Benjamini–Hochberg FDR significance threshold with a cut-off value of 0.05.

A study of potential EV protein biomarkers was conducted based on Gryciuk’s protocol [9], examining proteins shared by the B0399, WUT216, and WUT240 strains that are not homologous to human proteins. First, the peptide hits for each protein were summed up and sorted in ascending order for each strain (the assumption being that the quality of the biomarker depends on the relative frequency of peptide counts). To make the results independent of the total number of peptide counts per strain, the data were ranked using the following formula: rang = number of proteins × (number of unique scores remaining/total number of unique scores). Next, *K. marxianus* protein IDs were mapped to their corresponding standard names and then—using the NCBI database—to the NCBI gene ID. These IDs were then used to obtain the protein sequences in FASTA format (faa) from the NCBI database. Then, a BLASTP search was performed to identify proteins homologous to human proteins using the local UNIPROT human_reviewed database, with the following high-pass parameters: max sequences = 5, e-value = 10. Then, the homologs were flagged as BLAST results with e-value < 10^−3^ and coverage ≥ 50.

### 4.5. Human Cell Lines Cultivation

Five adherent intestinal epithelial cell lines were used—three cancer cell lines (HCT 116 (ATCC CCL-247), HT-29 (ATCC HTB-38), and Caco-2 (ATCC HTB-37)) and two normal ones (CCD-18Co (ATCC CRL-1459) and CCD 841 CoN (ATCC CRL-1790)). The HT-29 and HCT 116 cells were grown in McCoy’s 5A medium (VWR International, Leuven, Belgium) supplemented with 10% fetal bovine serum (FBS, Columbia origin, EURx, Gdańsk, Poland) and 1% penicillin–streptomycin (pen/strep, Gibco, Waltham, MA, USA). The Caco-2 cells in MEM medium (Sigma-Aldrich, St. Louis, MO, USA) were supplemented with 20% FBS, 1% pen/strep, and 0.1 mM NEAA (Sigma-Aldrich, St. Louis, MO, USA), and the normal cells in MEM medium (VWR International, Leuven, Belgium) were supplemented with 10% FBS, 1% pen/strep, and 2 mM L-glutamine (Gibco, Waltham, MA, USA). The growth conditions were as follows: 37 °C and 5% CO_2_. After reaching ~80% confluence, the cells were passaged according to the following protocol: washed with PBS, trypsinized with 0.25% trypsin–EDTA (Gibco, Waltham, MA, USA) for ~5 min at 37 °C, 5% CO_2_, and then neutralized with the appropriate medium. Detailed protocols for cell seeding are set in the descriptions of the methods used in the study, which are outlined in the following text.

### 4.6. Cytotoxicity Tests—MTT and ROS Assays

MTT and ROS assays were performed to evaluate the effects of *K. marxianus* EVs on human colon epithelial cells. In total, 100 µL of either cancer (HCT 116, HT-29, Caco-2) or normal (CCD-18Co, CCD 841 CoN) cells, at concentrations of 5 × 10^4^ cells/mL or 2.5 × 10^4^ cells/mL, respectively, were seeded onto a 96-well plate and incubated for 24 h at 37 °C with 5% CO_2_. The lower concentration of normal cells was used due to their larger cell size compared with cancer cells. On the second day, EV suspensions were prepared in full medium (McCoy’s 5A or MEM) at concentrations of 1 × 10^2^, 1 × 10^3^ or 1 × 10^4^ EVs per cell. The subsequent steps depended on the method used and are described below.

*MTT assay*. The medium was discarded from the wells. Then, 100 µL of EV suspensions, pure medium (negative control), or 5% dimethyl sulfoxide (DMSO, positive control, Thermo Scientific, Waltham, MA, USA) was added directly to the wells. The plates were then incubated for either 24 or 48 h at 37 °C and 5% CO_2_. Next, the liquid was removed, and 100 µL of the MTT solution (3-(4,5-dimethyl-2-thiazolyl)-2,5-diphenyl-2H-tetrazolium, Sigma-Aldrich, St. Louis, MO, USA) prepared in a suitable medium at a concentration of 0.5 mg/mL (for cancer lines) or 1 mg/mL (for normal lines) was added. The plates were incubated for 2 h under standard conditions. The MTT solution was then removed, and 50 µL of 100% DMSO was added to each well. The plates were shaken for 10 min, and the absorbance was measured at 570 nm using a Synergy H4 Hybrid plate reader (BioTek Instruments, Winooski, VT, USA). Metabolic activity (MA) was calculated using the following formula: %MA = (A_sample − A_blank)/(A_mean_negative_control − A_blank) × 100%.

*ROS (Reactive Oxygen Species) assay*. The medium was discarded from the wells, and then 100 µL of a freshly prepared dcFDA solution (2′,7′-dichlorodihydrofluorescein diacetate; Sigma-Aldrich, St. Louis, MO, USA) in a suitable medium was added to each well. The plates were incubated for 1 h in the dark at 37 °C with 5% CO_2_. The dcFDA solution was then removed, and 100 µL of the EV suspension, pure medium (negative control), 100 µM t-BHP (tert-butyl hydroperoxide, oxidant; Sigma-Aldrich, St. Louis, MO, USA), or 5 mM GSH (glutathione, antioxidant; Sigma-Aldrich, St. Louis, MO, USA) was added directly to the wells. The plates were then incubated for an additional 24 h under standard conditions. The fluorescence intensity was read at 485 ± 9/535 ± 9 nm (excitation/emission) using a plate reader. Relative ROS production level was calculated using the following formula: ROS = F_sample/F_mean_negative_control × 100%, where F means fluorescence intensity.

### 4.7. Assessment of Interleukin-8 Levels in the HT-29 Human Cell-Line Cultures

The immunological response of the HT-29 cell line to yeast EVs was assessed, with a focus on the pro-inflammatory interleukin-8 (IL-8). The cells were cultured in McCoy’s 5A medium containing 10% heat-inactivated FBS (incubated for 30 min at 56 °C) and 1% pen/strep [31]. First, 1 mL of human cells at a conc. of 1 × 10^6^ cells/mL was seeded into a 12-well microplate and incubated for 24 h at 37 °C and 5% CO_2_. Then, the cells were washed with PBS and treated with 1 mL of EVs at the specified conc. of 1 × 10^3^ EVs/cell for 24 h under the same conditions. PBS was used as a negative control, and LPS at a conc. of 100 ng/mL was used as a positive control. After incubation, the cells were detached by trypsinization and centrifuged. The resulting supernatant (100 µL) was used in the Human CXCL8/IL-8 Sandwich ELISA Kit (Proteintech Group, Rosemont, IL, USA) according to the manufacturer’s protocol.

### 4.8. Immunodetection of IL-8-like Protein and TNF-α-like Protein in Hemocytes of Galleria Mellonella Larvae

A colony of *G. mellonella* wax moths (Lepidoptera: Pyralidae) was maintained in chambers with controlled temperature and humidity (30 °C, 70% r.h.) in constant darkness (without photoperiod). Larvae were fed an artificial diet prepared according to Sehnal’s recipe, containing wheat flour, wheat bran, corn flour, skimmed milk powder, honey, and glycerin. Hemolymph was then collected from larvae on the first day of the last larval instar. To sterilize the larval surface and minimize sample contamination, larvae were first washed with 70% (*v*/*v*) ethanol and then briefly rinsed with distilled water. Hemolymph was then collected through an incision made in the last proleg.

To establish hemocyte cultures, hemolymph was adjusted to a hemocyte density of 2 × 10^4^ cells/mL and combined with supplemented Grace’s Insect Medium (GIM; Invitrogen, Carlsbad, CA, USA) at a hemolymph: GIM ratio of 1:3 (*v*/*v*). The GIM was supplemented with gentamicin (10 mg/mL; Gibco), amphotericin B (250 μg/mL; Gibco), and phenylthiourea (PTU; 0.1 mM; Sigma-Aldrich) to prevent melanization. Depending on the experimental group, the hemolymph–GIM suspension received no further addition (untreated, Control 1) or additionally contained PBS (Dulbecco’s PBS, 1×, w/o Ca and Mg, w/o phenol red, sterile-filtered; Capricorn Scientific; Control 2), LPS at a final concentration of 100 ng/mL in hemolymph (lipopolysaccharide from *Salmonella typhimurium*; Calbiochem/Merck Millipore; Control 3), or EVs of *K. marxianus* strain WUT240 or WUT216 grown in SAB medium and suspended in PBS (30 μL of EVs suspension added to the 300 μL of hemolymph–GIM mixture, 1 × 10^5^ EVs per hemocyte). The suspension (100 μL per well) was seeded into six-channel μ-Slide IV 0.4 chambers (Ibidi, Gräfelfing, Germany), and the entire procedure was performed in three independent biological replicates. Samples were incubated at 27 °C for 1.5 h.

Following incubation, hemocytes were fixed with 4% paraformaldehyde (PFA; Sigma-Aldrich) and permeabilized with 0.1% Triton X-100 (Sigma-Aldrich), both prepared in PBS (phosphate-buffered saline tablets; Invitrogen). Non-specific antibody binding was blocked by incubation in 4% BSA-PBS for 1 h. Cells were then incubated overnight at 4 °C with rabbit polyclonal primary antibodies against IL-8 (ab106350, Abcam) or TNF-α (ab6671, Abcam, Cambridge, UK), diluted 1:60 in PBS. After washing, samples were incubated for 2 h at room temperature with goat anti-rabbit IgG (H + L) DyLight 488-conjugated secondary antibody (Invitrogen) at a concentration of 2 μg/mL. Actin filaments were counterstained with ActinRed 555 ReadyProbes Reagent (Invitrogen), and cell nuclei were stained with NucBlue™ Fixed Cell ReadyProbes™ Reagent (Invitrogen). Fluorescence signals were visualized using an Axiovert 5 fluorescence microscope (Zeiss, Oberkochen, Germany) equipped with an Axiocam 305 color digital camera (Zeiss). Images were acquired and analyzed using ZEN 3.2 lite software with the Image Analysis Module (Zeiss). Each experimental condition was assessed in three independent biological replicates. For each image, the mean fluorescence intensity of the green channel (corresponding to the IL-8-like or TNF-α-like signal) was measured using the Intensity Mean Value feature of the Image Analysis Module in ZEN 3.2 lite software (Zeiss). This value was corrected by subtracting the mean background intensity, measured in a cell-free region of the same image, yielding the corrected fluorescence intensity for each image. Corrected values are expressed in arbitrary units (a.u.)—i.e., raw pixel gray-scale values, whose numerical range depends on the camera’s bit depth and image acquisition settings and which are therefore meaningful only for relative comparison among samples imaged under identical conditions, not as an absolute physical unit.

### 4.9. Yeast EV Interaction with Human Intestinal Cell Lines

Microscopic observations were conducted to investigate the interaction between *K. marxianus* EVs and human colonic epithelial cells (HCT 116, HT-29, Caco-2, CCD-18Co, and CCD 841 CoN). The EVs isolated from yeast cultures in SAB and SCF were stained with Nile Red (NR) as previously described [8]. First, 100 µL of EVs at a conc. of 1 × 10^11^ EV/mL were incubated with 4 µL of a 2.5 mg/mL Nile Red solution (Carl Roth, Karlsruhe, Germany) diluted in PBS for 30 min in the dark at RT. At the same time, a sample containing only PBS instead of the EVs volume was used as a control for the subsequent washing steps. Then, 300 µL of PBS was added, and the solution was transferred to filter columns with a 10 kDa cut-off (Merck, Darmstadt, Germany). Eight rounds of centrifugation (14,000× *g*, 5 min, RT) and washing of the condensate were performed using 400 µL of PBS each time. The condensate was then transferred to a fresh tube. The samples were stored protected from light. A culture of human intestinal cells was set up at a concentration of 3.13 × 10^4^ cells/mL and incubated for 72 h at 37 °C with 5% CO_2_ on the microscopic slides with 8-well chambers for cell culture (Biologix Group Ltd., Jinan, China). The medium was then discarded, and the wells were gently washed three times with PBS. After that, 15 µL of NR-stained EVs, control PBS, or pure PBS were added to the wells and incubated for 15 min in the dark under standard conditions. The sample solutions were discarded. Then, 200 µL of 3.7% paraformaldehyde (PFA, Sigma-Aldrich, St. Louis, MO, USA) solution was added and incubated for 20 min at RT in the dark. The PFA was removed, and the cells were gently washed twice with PBS. To stain the cell nuclei, 200 µL of 300 nM DAPI solution (4′,6-diamidino-2-phenylindole, Sigma-Aldrich, St. Louis, MO, USA) in PBS was added to the cells and left for 5 min at RT in the dark. Then, the wells were gently washed once with PBS. Microscopic observation was performed using a Nikon Eclipse Ni fluorescence microscope, with Plan Fluor 60×/0.85, CFI 10×/22 ocular, blue (392/23 nm; 447/60 nm) or red (554/23 nm; 609/54 nm) fluorescent filters, and a Nikon DS-Fi3 camera (Nikon Instruments, Tokyo, Japan). Images were captured using NIS Elements DR ver. 5.30.04 software (Nikon Instruments, Tokyo, Japan).

### 4.10. Encapsulation of Doxorubicin into Yeast EVs and Their Application to Human Cell Lines

EVs were collected from yeast cultures in SAB and loaded with doxorubicin (DOX, doxorubicin hydrochloride, Pol-Aura, Zabrze, Poland), as described previously [8]. First, 450 µL of EVs at a conc. of 1 × 10^11^ EV/mL were incubated with 50 µL of 4.5 mM doxorubicin in PBS for 30 min at 4 °C in the dark. Simultaneously, a sample containing only PBS without EVs was used as a control for the subsequent rinsing steps. Then, 500 µL of a 400 mM sucrose solution (dissolved in PBS and filtered using a 0.1 µm membrane filter (AppliChem, Darmstadt, Germany)) was added to each sample, and the samples were incubated for a further 20 h at 4 °C. The samples were transferred into filter columns with a 10 kDa cut-off (Merck, Darmstadt, Germany) and centrifuged (14,000× *g*, 15 min, RT). The retentate was then washed with 400 µL of PBS by spinning under the same conditions. This procedure was repeated five times to ensure that any unbound doxorubicin was removed from the sample. To release the EVs, 400 µL of PBS was added to the filter column. Then, the column was inverted, placed in a new tube, and centrifuged (1000× *g*, 2 min, RT). The samples were stored protected from light. Caco-2 cells, seeded at a conc. of 4 × 10^4^ cells/mL, were incubated for 72 h at 37 °C with 5% CO_2_ on the microscopic slides with 8-well chambers for cell culture. The medium was then discarded, and the wells were gently washed three times with PBS. Then, 100 µL of EVs with or without loaded DOX and control PBS with or without DOX, along with 100 µL of medium, were added to the wells and incubated for 1 h at 37 °C, 5% CO_2_. Subsequent washing, fixation, permeabilization, and DAPI staining procedures were performed as described above, and images were acquired using confocal microscopy (Olympus Fluoview FV10i, Tokyo, Japan).

### 4.11. Statistical Analysis

Experiments were performed with three independent biological replicates (*n* = 3), each comprising one to five technical replicates. Data were expressed as the mean ± SE unless stated otherwise; statistically significant differences were determined using one-way ANOVA analysis with Tukey’s HSD post hoc test. For MTT and ROS data, statistical comparisons were performed between treated and untreated cells. For IL-8 in the HT-29 cell-line culture, statistical analysis was performed using PBS control as the reference group. Data were analyzed using Origin Pro 2021b software v9.8 with an academic license (OriginLab Corporation, Northampton, MA, USA). For the study of *G. mellonella* hemocytes, statistical comparisons among the five experimental groups (Control 1, Control 2, Control 3, WUT216, and WUT240) were performed separately for each cytokine using one-way ANOVA analysis, followed by the Tukey HSD post hoc test for pairwise comparisons, using STATISTICA 6.1 software (StatSoft Polska, Kraków, Poland).

## 5. Conclusions

This study proved the ability of the *K. marxianus* B0399, WUT240, and WUT216 strains to release EVs, with production efficiencies of approximately 10^10^ EVs/mL in SAB medium. In addition, *K. marxianus* also produces EVs in simulated human gastrointestinal fluids in the stomach, small intestine, and large intestine; however, it does so with slightly lower productivity than in SAB. Importantly, yeast vesicles are non-toxic to normal human intestinal cell lines, neither negatively affecting metabolic activity nor ROS levels, and even exhibit mild antioxidant properties. The immunogenicity investigation underlines safety, demonstrating no induction of the pro-inflammatory chemokine IL-8 in the late response of the HT-29 cell line. Moreover, *K. marxianus* EVs are able to interact with human epithelial cells in vitro and deliver bioactive molecules, such as doxorubicin, to them. Of course, further research on their effects in higher model organisms is needed, including the delivery of their own cargo (proteins, DNA, and RNA) and immunogenicity.

## Figures and Tables

**Table 1:** Particle size characterization of EVs released by *K. marxianus* in fluids mimicking the gastrointestinal tract environment (SGF, SIF, and SCF). For each culture variant, a corresponding control in SAB-based medium was used. No significant differences were observed (mean ± SE, *n* = 3, one-way ANOVA with the Tukey HSD post hoc test, *p* > 0.05).

Strain	Time (h)	Medium	Diameter Mode (nm)	Diameter Mean (nm)
B0399	1	control	79.8 ± 2.96	111.7 ± 3.18
		SGF	91.5 ± 11.02	200.8 ± 33.63
	2	control	93.2 ± 10.97	141.3 ± 17.13
		SIF	73.6 ± 12.85	135.0 ± 5.93
	24	control	94.8 ± 14.35	136.7 ± 2.85
		SCF	100.2 ± 3.67	123.0 ± 3.46
WUT240	1	control	80.0 ± 7.50	131.0 ± 46.00
		SGF	110.5 ± 20.64	194.0 ± 37.16
	2	control	125.5 ± 26.06	160.0 ± 28.29
		SIF	135.8 ± 8.41	351.0 ± 23.18
	24	control	137.2 ± 2.03	168.7 ± 0.88
		SCF	121.8 ± 3.46	151.0 ± 9.02
WUT216	1	control	98.8 ± 4.48	129.0 ± 3.21
		SGF	97.5 ± 5.77	160.7 ± 4.67
	2	control	106.0 ± 4.72	137.0 ± 2.08
		SIF	113.5 ± 15.39	245.7 ± 18.9
	24	control	122.5 ± 1.00	147.7 ± 1.20
		SCF	111.5 ± 5.51	151.0 ± 9.02

## Data Availability

The MS data were deposited at https://repository.jpostdb.org/mypage under project number JPST004863 [18] and are available via ProteomeXchange with dataset identifier PXD082951.

## Supplementary Materials

The following supporting information can be downloaded at https://www.mdpi.com/article/10.3390/ijms27209259/s1.
