# Supplementary material for: Characterization of Kluyveromyces marxianus Extracellular Vesicles as a New Potential Delivery System for Biologically Active Compounds
Source: Int J Mol Sci. 2026 Oct 18;27(20):9259. doi: 10.3390/ijms27209259 (PMC13705037; doi:10.3390/ijms27209259)
Supplement: Supplementary file 1 [file ijms-27-09259-s001.zip › Supplementary material_Figure S1.pdf]

Characterization of *Kluyveromyces marxianus* Extracellular Vesicles as a New Potential Delivery System for Biologically Active Compounds

Marta Rogalska [1], Katarzyna Kmita [1], Anna Katarzyna Wrońska [2], Aleksander Gryciuk [1], Ewa Sitkiewicz [3], Magdalena Bakun [3], Magdalena Świadek [4], Małgorzata Milner-Krawczyk [1] and Jolanta Mierzejewska [1,*]

* Correspondence: jolanta.mierzejewska@pw.edu.pl

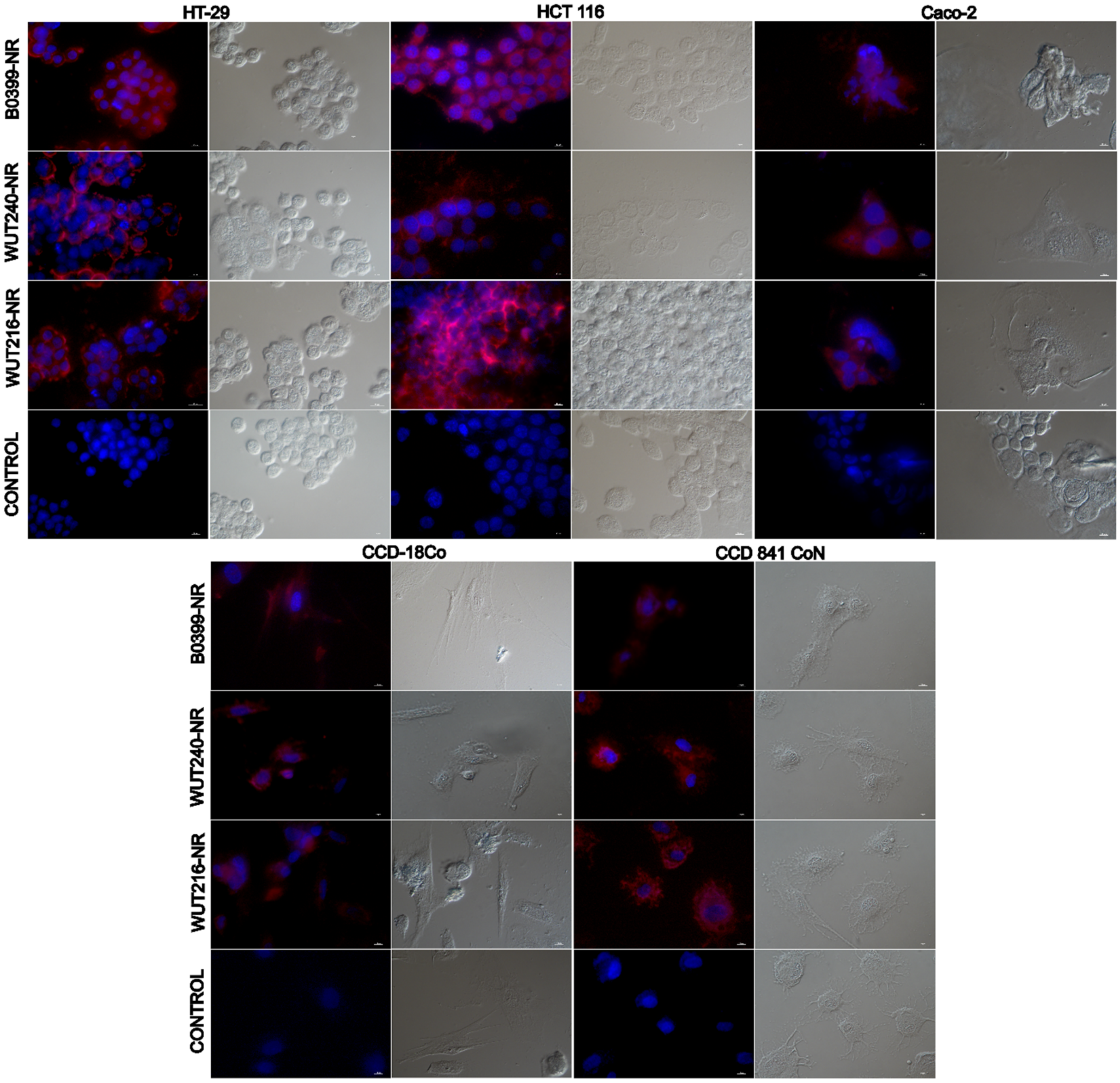

Figure S1. Internalization of *K. marxianus* EVs (SCF) with human cancer and normal cell lines. EVs were stained with red fluorescence Nile Red dye. PBS control lacking EVs subjected to identical staining and washing steps. Cellular nuclei were stained with DAPI. The scale bar is 10 μm.

To complement the research, an investigation was also carried out into the interaction of yeast EVs (SCF) with human intestinal cells under in vitro conditions (Figure S1). The EVs were stained with Nile red dye and then added to the cell culture. The presence of EVs (red fluorescence) was observed around the cell nucleus (blue fluorescence). Furthermore, no red fluorescence was visible in the intercellular spaces, which additionally confirmed the direct interaction of yeast EVs with human cells.
